# Ablation with zero‐fluoroscopy of premature ventricular complexes from aortic sinus cusps: A single‐center experience

**DOI:** 10.1002/joa3.12642

**Published:** 2021-10-03

**Authors:** Pablo J. Sánchez‐Millán, Guillermo Gutiérrez‐Ballesteros, Manuel Molina‐Lerma, Rosa Macías‐Ruiz, Juan Jiménez‐Jáimez, Luis Tercedor, Miguel Álvarez

**Affiliations:** ^1^ Arrhythmia Unit Hospital Universitario Virgen de las Nieves Granada Spain; ^2^ Instituto de investigación biosanitaria de Granada (FIBAO) Granada Spain

**Keywords:** aortic sinus cusp ventricular arrhythmias, intracardiac echocardiography, zero‐fluoroscopy

## Abstract

**Background:**

Catheter ablation of premature ventricular complexes from aortic sinus cusps (ASC‐PVC) is a complex procedure that conventionally requires coronary catheterization (CC) to localize coronary artery ostium (CAO). Little published information is available on the mapping and ablation with zero‐fluoroscopy (ZF) of ASC‐PVC. The aim of the study was to determine the efficacy and safety of ASC‐PVC ablation with a ZF approach guided by 3D intracardiac echocardiography integration in the electroanatomical mapping system (ICE 3D‐EAM).

**Methods:**

This observational study included one patient cohort treated conventionally and another treated with ICE 3D‐EAM‐guided ZF ablation. Clinical, efficacy, and safety outcomes were evaluated acutely and at 3 months follow‐up.

**Results:**

The study included 21 patients with ASC‐PVC: 10 in the ZF group (age 49 ± 16 years, 60% males) and 11 in the control group (age 47 ± 15 years, 27% males). Fluoroscopy was not required for any patient in the ZF group. Acute success was obtained in 80% of the ZF group vs 55% of the control group (*P* = .36). The recurrence rate was 30% in the ZF group vs 27% in the control group (*P* = 1). One nonsevere complication was observed in the ZF group (*P* = .48).

**Conclusions:**

ZF catheter ablation of ASC‐PVC guided by ICE 3D‐EAM is feasible, effective, and safe.

## Graphical abstract

The graphical abstract shows the workflow for ZF ablation approach of ASC‐PVC using ICE 3D‐EAM. The key point is the ICE 3D‐EAM reconstruction which allows to assess the distance of the ablation catheter tip to CAO. Note that if the distance is at least >5 mm from earliest LVAT to CAO, the ablation is started. On the other hand, with a distance <5 mm respect to CAO, the ablation is not performed and another safe adjacent site is searched. ZF: zero‐fluoroscopy. ASC: Aortic sinus cusp. PVC: Premature ventricular complex. LV: Left ventricle. RV: Right ventricle. CAO: Coronary artery ostium. LVAT: Local ventricular activation time. ICE 3D‐EAM: Electroanatomic reconstruction guided by intracardiac echocardiography.

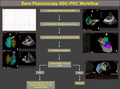

**Abbreviations**
AAD: antiarrhythmic drugs.
ACT: activation clotting time.
ASC‐PVC: premature ventricular complex from aortic sinus cusp.
CA: coronary artery.
CAO: coronary artery ostium.
CC: coronary catheterization.
ECG: electrocardiogram.
EGM: electrogram.
EPS: electrophysiological study.
ICE 3D‐EAM: electroanatomic reconstruction guided by intracardiac echocardiography.
ICE: intracardiac echocardiography
LCC: left coronary cusp
LMCA: left main coronary artery
LVAT: local ventricular activation time
LVOT: left ventricular outflow tract
NCC: non‐coronary cusp
PVC: premature ventricular complex
RCA: right coronary artery
RCC: right coronary cusp
RF: radiofrequency
RVOT: right ventricular outflow tract
ZF: zero‐fluoroscopy

## 1 INTRODUCTION

Premature ventricular complexes (PVC) frequently originate from aortic sinus cusps (ASC‐PVC), commonly in patients with no structural cardiovascular disease.^1^ Their ablation is challenging due to their proximity to coronary artery ostium (CAO).^2^, ^3^, ^4^, ^5^ Coronary catheterization (CC) has conventionally been used to avoid complications during ablation and evaluate the distance from CAO. Over the past few years, there has been an increase in the implementation of zero‐fluoroscopy (ZF) ablation guided by electroanatomical mapping (EAM) systems and imaging techniques.^6^, ^7^, ^8^ However, little information is available on the outcomes of ablation with ZF in patients with ASC‐PVC.

The objective of this study was to compare safety and efficacy outcomes between patients with ASC‐PVC treated with ZF ablation guided by 3D integration of intracardiac echocardiography images in the EAM (ICE 3D‐EAM) and those treated with the conventional approach.

## 2 METHODS

### 2.1 Study population, design, and patient selection

This observational, retrospective, and single‐center study consecutively recruited patients undergoing ASC‐PVC ablation at our center. Among 37 patients who underwent 3D ICE‐EAM‐guided PVC ablation with ZF between April 2019 and September 2020, 10 (27%) with origin in ASC were enrolled in the ZF group. Among 56 patients with PVC who underwent a conventional approach between July 2015 and March 2019, 11 (19.6%) with ASC‐PVC who required fluoroscopy and CC but not ICE were enrolled in the control group. The study was approved by the local ethics committee, and all participants signed their informed consent.

### 2.2 Study protocol and follow‐up

The procedure was performed without general anesthesia after the suspension of antiarrhythmic drugs (AADs) for at least five half‐lives. The ablation protocol for each group is detailed below. All patients underwent electrophysiological study (EPS) with the LabSystem Pro™ recording system (Boston Scientific*;* bipolar filter 30‐250 Hz, unipolar Filter: 1‐250 Hz, electrocardiogram [ECG] filter 1‐100 Hz; sweep speed: 100 mm/s). Two operators with experience in PVC ablations and ICE‐guided procedures performed all interventions. Local ventricular activation time (LVAT) was measured from onset of bipolar electrogram (EGM) in the ablation or mapping catheter until onset of QRS. Pace‐mapping was performed at 2 mA above of diastolic threshold and a pacing cycle length of 600 ms After accessing the arterial circulation, unfractionated heparin was administered to maintain an activation clotting time >250 s.

Success of the procedure was defined by complete PVC elimination baseline and after isoprenaline administration (0.5‐2.0 mcg/min) at the end of a 30 minute waiting period. All patients underwent 24 hours Holter monitoring at 3 months post‐ablation. Success during the follow‐up was defined by a reduction of >80% in baseline PVC density and by the absence of AADs to control symptoms not present before the ablation.

Data were gathered and analyzed on the baseline characteristics of the two groups, procedure‐related safety and efficacy variables, and the presence/absence of recurrence during a 3 month follow‐up period.

### 2.3 Approach in the ZF group guided by ICE 3D‐EAM

**FIGURE 1:**
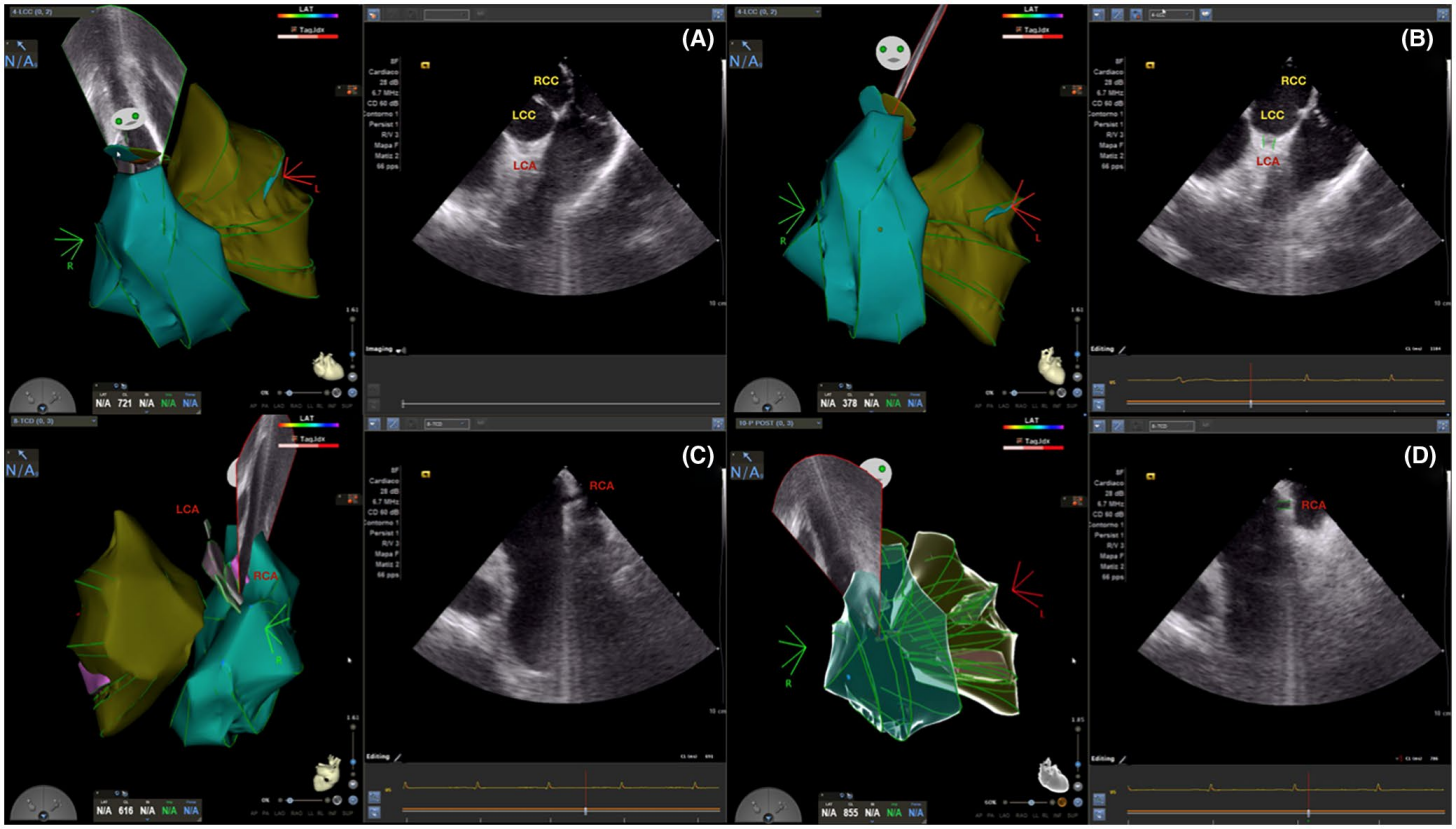
Identification of CAO localization and its integration with ICE 3D‐EAM (see text for a detail explanation). (A, B) Left CAO localization and ICE 3D‐EAM integration. (C, D) Right CAO localization and ICE 3D‐EAM integration. LCC: Left coronary Cusp. RCC: Right Coronary Cusp. LCA: Left Coronary Artery. RCA: Right coronary artery. CAO: Coronary artery ostium

CARTO® navigation system (Biosense Webster) was used for all patients. All vascular accesses were obtained by ultrasound guidance. One femoral venous access was cannulated for the ICE catheter and, in cases requiring initial examination of the right ventricular outflow tract (RVOT), another for the ablation or mapping catheter. The ICE catheter (Soundstar Biosense Webster) was advanced to the right atrium under visualization with the Acuson X300™ ultrasound system (Siemens Medical Solutions USA, Inc) (Appendice material 1 and video S3). Next, beginning from the Homeview, the cardiac cavities of interest (right ventricle, RVOT, pulmonary valve, left ventricle, left ventricular outflow tract [LVOT], ASC, and CA ostia) were reconstructed and integrated using CARTOSOUND® (Biosense Webster) (Graphical abstract). This module outlines the ultrasound image of each structure under study and generates a 3D reconstruction of the cavities and large vessels that is integrated in the CARTO® system. The left main coronary artery (LMCA) was localized on the view of the aortic valve plane, where it emerges at 5‐6 o'clock from the left coronary cusp (LCC) (Figure 1A,B; Video S1). The origin of the right coronary artery (RCA) was found by positioning the ICE catheter in the right atrial appendage with mild anteflexion or by unfolding the ascending aorta from the RV in a position close to the ICE catheter tip at around 12 o'clock (Figure 1C,D; Video S1). Both arteries were reconstructed from their origin to the first 2‐3 cm of their trajectory. After the anatomic reconstruction, the activation map was generated using the Thermocool Smart‐Touch® ablation catheter (Biosense Webster) or PentaRay® catheter (Biosense Webster) according to the operator's criteria. A direct approach to the LVOT was performed unless the PVC morphology showed a transition in V3 or beyond, when an initial RVOT approach was selected. Aortic retrograde LVOT approach was performed via right femoral artery if RVOT activation mapping was not appropriate (absence of QS in unipolar, LVAT in bipolar EGM to QRS onset <20 ms, and/or pace‐mapping <90%) or if ablation was not successful at the point of earliest LVAT. The ZF approach to the LVOT via retrograde transaortic access is described in the Appendice material 2 and video S4.

For the passage of the aortic valve with the ablation catheter, we place the catheter posteriorly, in the noncoronary cusp (NCC) or between de NCC and the right coronary cusp (RCC), with the deflection (red label in the tip catheter) facing forward. After this, we deflect the catheter to form a pigtail while monitoring not to introduce the tip into the CAO, previously located and defined with CARTOSOUND®. Once we have made a pigtail with the ablation catheter the passage of the aortic valve is simple with torque and light pressure (video S1 in supplementary material).

**FIGURE 2:**
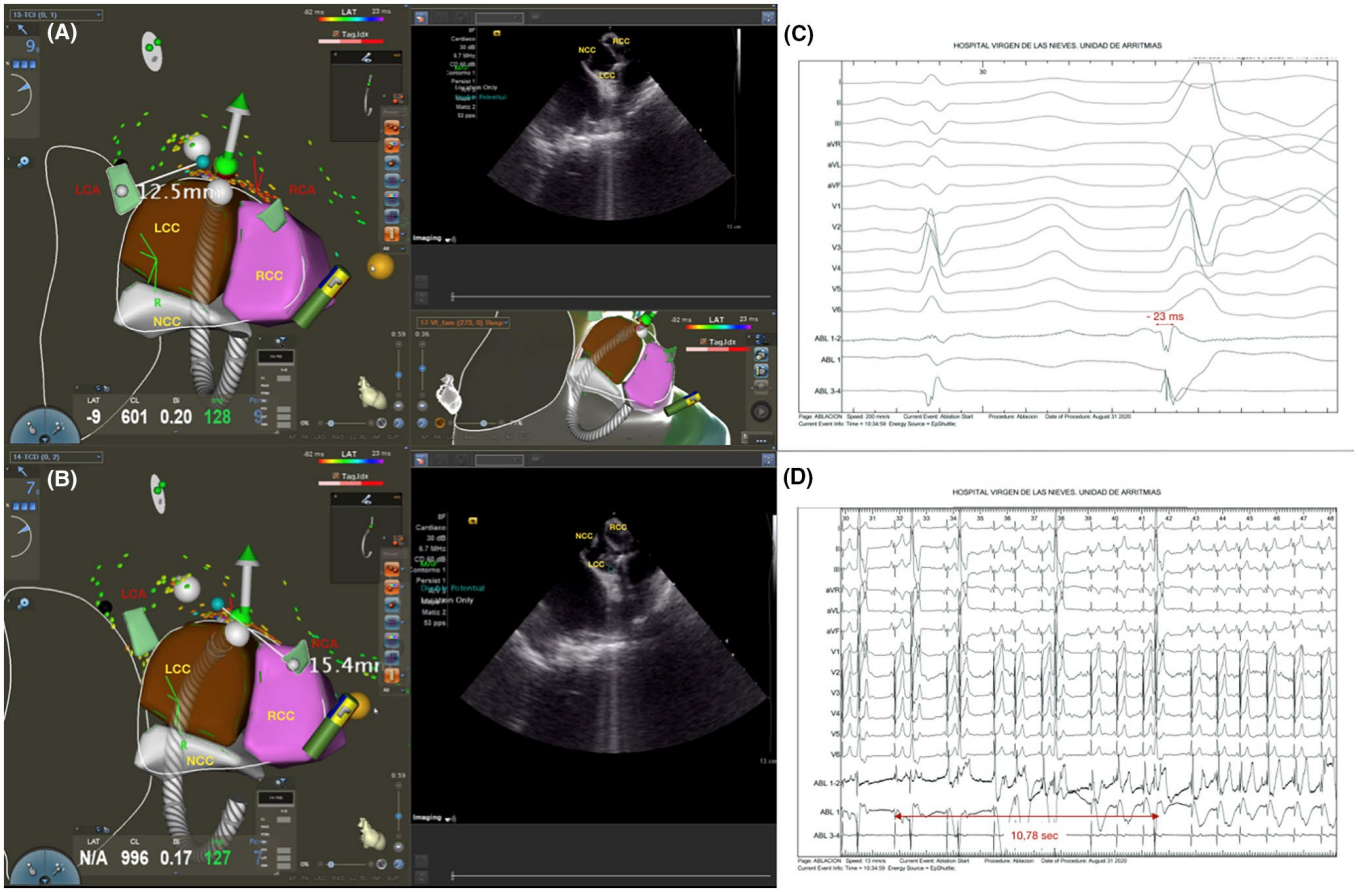
PVC ablation with origin in LCC‐RCC using ICE 3D‐EAM guidance. (A, B) The successful ablation spot was located 12.5 mm to left CAO and 15.4 to right CAO. The ICE 3D‐EAM reconstruction allowed to perform an ablation with a safe distance to CAO. C: EGM at the successful ablation site. D: Start of RF ablation and PVC suppression after 11 seconds. LCC: Left coronary Cusp. RCC: Right Coronary Cusp. NCC: Noncoronary Cusp. LCA: Left Coronary Artery. RCA: Right coronary artery. CAO: Coronary artery ostium. EGM: Electrogram

**FIGURE 3:**
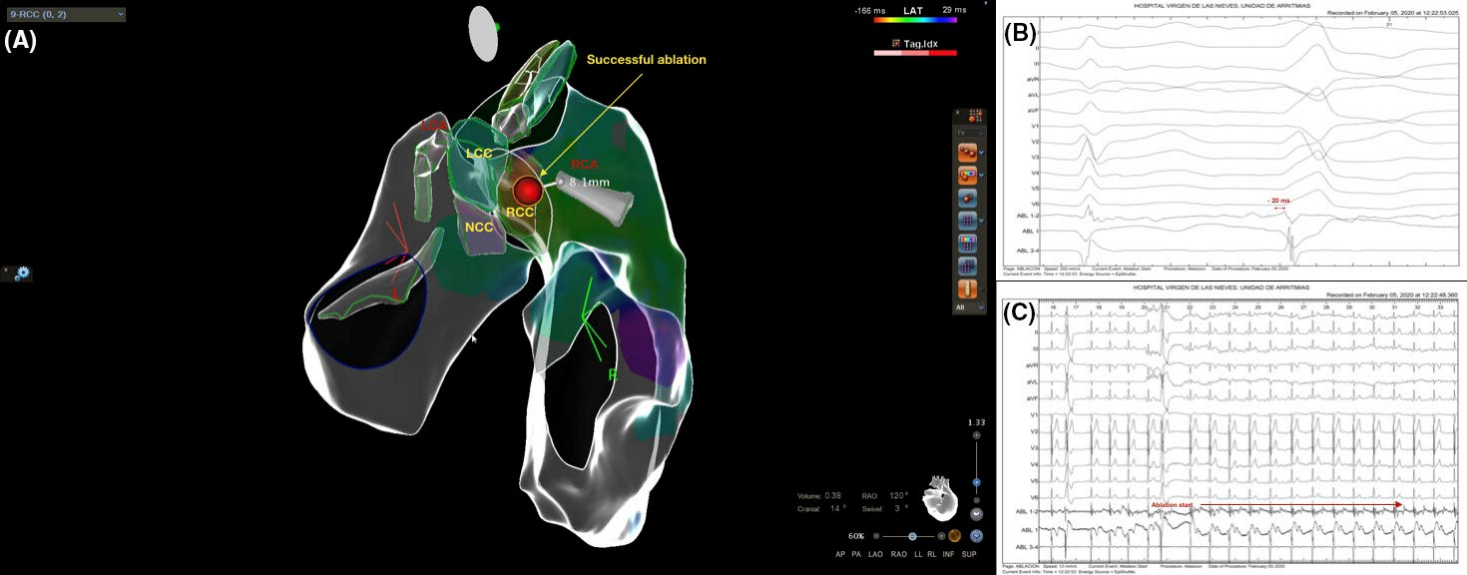
PVC ablation with origin in RCC. (A) The successful ablation spot was found in a safety localization respect to right CAO (distance of 8.1 mm) assessed with ICE 3D‐EAM. PVC was abolished in that site without any clinical or electrocardiographic alteration. (B) EGM at the successful ablation site. (C) Start of RF ablation and PVC suppression immediately. LCC: Left coronary Cusp. RCC: Right Coronary Cusp. NCC: Noncoronary Cusp. LCA: Left Coronary Artery. RCA: Right coronary artery. CAO: Coronary artery ostium

The distance from ablation catheter tip to the corresponding CAO, as visualized by both ICE and navigation system, was measured using the previous 3D reconstruction (Figure 2 and Figure 3). Radiofrequency (RF) was applied when the distance was >5 mm. During ablation, the catheter tip was continuously monitored using the navigation system and real‐time ICE visualization. When applied, RF was started at 20 W with contact of ≥5 g, upper temperature limit of 40℃, and irrigation flow of 17 mL/min. In cases of an adequate impedance drop (8‐10 Ω) or PVC suppression, RF was increased to a maximum of 40 W and 180 seconds per application (video S2). Postablation CC was not performed if no ST segment alterations or angina symptoms were observed during or after the ablation.

### 2.4 Approach in the control group

PVC activation mapping was performed using the Carto3® navigation system or EnSite Navx® (St Jude Medical). Before 2016, electroanatomical reconstruction was done using a Thermocool SF Nav ablation catheter (Biosense Webster) or PentaRay® catheter with the Carto3® system, or a Tacticath catheter (St Jude Medical) with the EnSite Navx® system, according to the operator's criteria. From 2016 onward, the Thermocool Smart‐Touch® catheter was used with the Carto3® system. The order of cardiac cavities mapping and the ablation criteria were the same as those for the ZF group.

After locating the earliest LVAT in ASC, CC was performed in the corresponding coronary artery (CA) to measure the distance between ablation catheter tip and CAO, using orthogonal planes and taking the width of the ablation catheter as reference (2.5 mm). CC was carried out in one or both CAs according to the operator's criteria. RF was applied when the distance was >5 mm in at least one angiographic projection. Application parameters were the same as for the ZF group. During ablation, the position of the ablation catheter and the ST segment were monitored by fluoroscopy to evaluate CA involvement. Postablation CC was not performed when no ST segment alterations or angina symptoms were observed during or after ablation.

### 2.5 Statistical analysis

Continuous variables were expressed as mean ± standard deviation (SD) and categorical variables as absolute values and percentages. The Shapiro‐Wilk test was used to evaluate the normality of data distribution. Categorical variables were compared with Pearson's *X* ^2^ test or Fisher's exact test. Quantitative variables were analyzed with the Student's t test when their distribution was normal and with the Mann‐Whitney U test when it was not. SPSS version 21 (SPSS, IBM Corporation) was used for statistical analyses. A value of *P* < .05 was considered significant.

## 3 RESULTS

Table S1 display the clinical characteristics of the ZF group: 40% were females, mean age was 49 ± 16 years, mean left ventricular ejection fraction (LVEF) was 52 ± 11%, and 50% had cardiomyopathy. Indications for ablation were palpitations in 70% and low LVEF in 30%. Four patients had a history of failed ablation and eight were under treatment with β‐blockers. The mean PVC burden before EPS was 24.8 ± 7% (25 300 ± 10 090 PVC/ 24 hours). All ASC‐PVC showed left bundle branch block morphology, inferior axis, and transition ≤V3.

Results of procedure‐related variables are displayed in Table 1 for the ZF group and in Table S2 for the control group. In the ZF group, the mean procedure duration was 269 ± 56 minutes, ICE 3D‐EAM reconstruction time was 45 ± 14 minutes, activation mapping time was 64 ± 29 minutes, RF time was 324 ± 211 seconds, and there was a mean of 6 ± 5 applications per patient. PVCs most frequently originated from the junction between RCC and LCC (60%), followed by the junction between NCC and RCC (30%), and the RCC (10%). Ablation was successful in 80% of cases. In the ZF group, the mean distance from the successful ablation spot was 15 ± 6 mm to right CAO and 22 ± 7 mm to left main CAO. No electrocardiographic alterations or clinical symptoms compatible with coronary involvement were observed in any patients, and there was no need for CC due to a poor visualization of CAO. One patient had a mild pericardial effusion after RF application, but no intervention was required and the procedure was not suspended.

**TABLE 1:** Procedure‐related variables in the zero‐fluoroscopy group

	PVC ablation site	Mapping catheter/Ablation catheter	Bipolar earliest LVAT (ms)	Ablation success	Procedure time (min)	ICE 3D mapping time (min)	Activation mapping time (min)	RF ablation time (s)	Number of RF lesions	Right CAO distance (mm)	Left main CAO distance (mm)	Complications
Patient 1	LCC‐RCC	Smart‐Touch/Smart‐Touch	‒28	Yes	225	50	25	96	1	16	24	No
Patient 2	NCC‐RCC	PentaRay/Smart‐Touch	‒25	No	360	35	41	390	13	15	28	No
Patient 3	NCC‐RCC	Smart‐Touch/Smart‐Touch	‒30	Yes	285	44	114	170	2	25	33	No
Patient 4	LCC‐RCC	Smart‐Touch/Smart‐Touch	‒22	Yes	309	15	70	340	7	6	22	No
Patient 5	RCC	Smart‐Touch/Smart‐Touch	‒20	Yes	286	36	79	350	6	8	26	No
Patient 6	NCC‐RCC	PentaRay/Smart‐Touch	‒19	Yes	313	59	53	522	9	12	28	No
Patient 7	LCC‐RCC	Smart‐Touch/Smart‐Touch	‒18	Yes	245	46	99	190	3	23	17	No
Patient 8	LCC‐RCC	Smart‐Touch/Smart‐Touch	‒23	Yes	214	69	34	180	2	11	12	No
Patient 9	LCC‐RCC	Smart‐Touch/Smart‐Touch	‒26	Yes	169	43	43	202	1	14	21	No
Patient 10	LCC‐RCC	PentaRay/Smart‐Touch	‒19	No	285	48	81	801	18	20	9	Mild pericardial effusion

Abbreviations: CAO, Coronary artery ostium; LCC‐RCC between Right and Left coronary Cusp; LVAT, Local ventricular activation time; NCC‐RCC, between Right and Noncoronary Cusp; PVC, Premature Ventricular complex; RCC, Right Coronary Cusp; RF, Radiofrequency.

**FIGURE 4:**
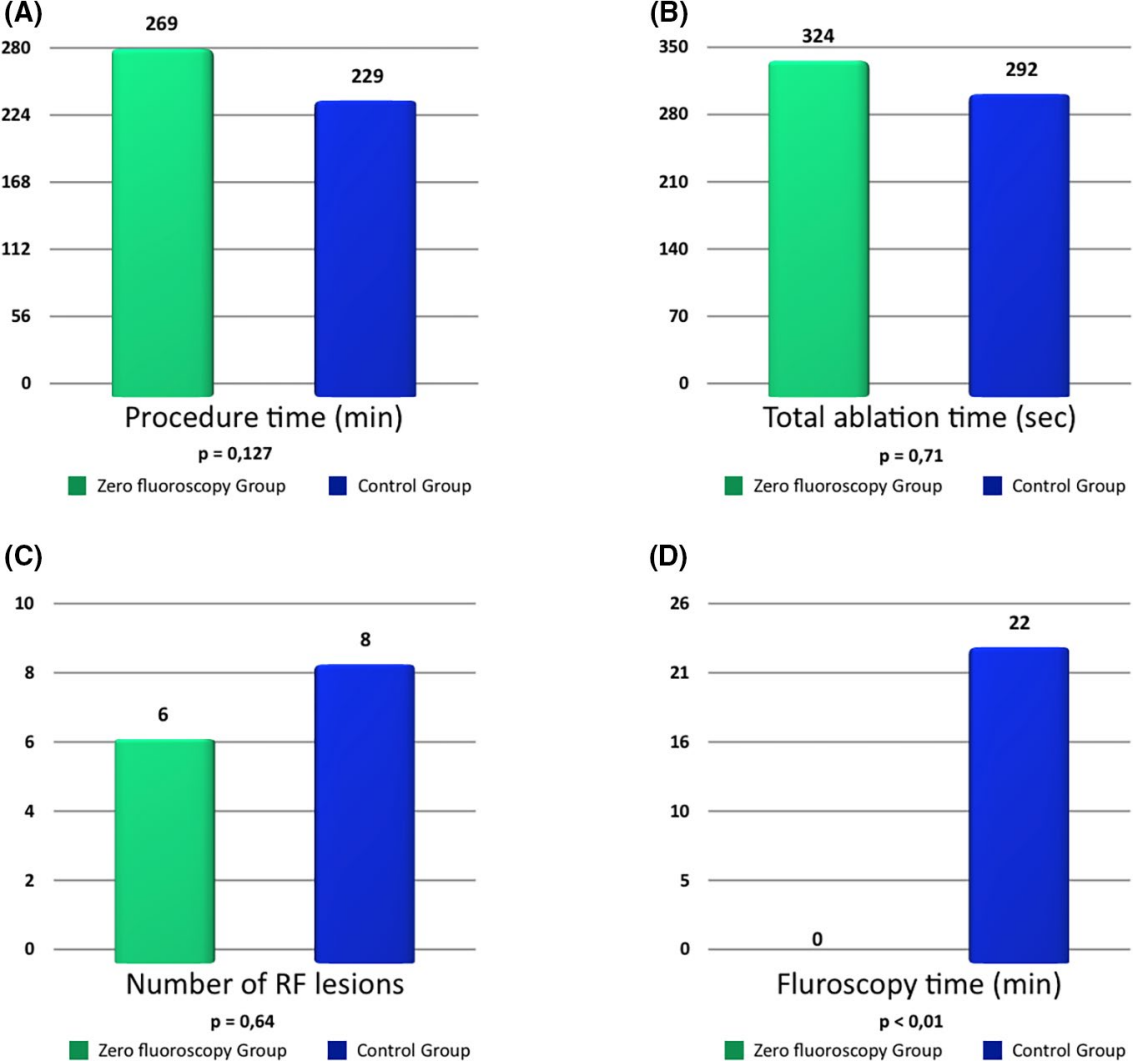
Differences in procedure characteristics between both groups. (A) Procedure time. (B) Total ablation time. (C) Number of RF pulses. (D) Fluoroscopy time. RF: Radiofrequency

Comparisons between the ZF and control groups are exhibited in Table 2. No significant between‐group differences were observed in baseline characteristics or in the following procedure‐related variables (Figure 4): procedure duration (269 ± 56 minutes for the ZF group vs 229 ± 60 minutes for the control group, *P* = .127), RF time (324 ± 211 seconds vs 292 ± 178 seconds, respectively, *P* = .71), number of applications (6 ± 5 vs 8 ± 7, respectively, *P* = .64), or complication rate (10% vs 0%, respectively, *P* = .48). No participant required postablation CC. Fluoroscopy time was significantly lower in the ZF vs control groups (0 min vs 22 ± 10 min, respectively *P* < .01). No significant between‐group difference was observed in rates of total acute success (80% in ZF group vs 55% in controls, *P* = .36) or success during 3 months follow‐up (70% vs 73%, respectively, *P* = 1).

**TABLE 2:** Comparison between the zero‐fluoroscopy group and control group

	Zero‐fluoroscopy group	Control group	Statistical significance
Age (y.o.)	49 ± 16	47 ± 15	*P* = .79 ^a^
Gender	40% Male	27% Male	*P* = .2^c^
	60% Female	73% Female	
Hypertension	20%	27%	*P* = 1^c^
Diabetes mellitus	10%	9%	*P* = 1^c^
Hyperlipidemia	10%	27%	*P* = .58^c^
Obesity (BMI >30)	30%	36%	*P* = 1^c^
Cardiomyopathy	50%	54%	*P* = 1^c^
LVEF (%)	52 ± 11%	47 ± 13%	*P* = .3^a^
PVC density prior to ablation (% and absolute number)	24.8 ± 7%	25.6 ± 7%	% *P* = .82^a^
	Absolute number: 25 300 ± 10 090	Absolute number: 34 813	Absolute number: *P* = .57 ^b^
Total procedure time (min)	269 ± 56	229 ± 60	*P* = .127 ^a^
Total ablation time (s)	324 ± 211	292 ± 178	*P* = .71 ^a^
Number of RF^1^ lesions	6 ± 5	8 ± 7	*P* = .64 ^b^
Fluoroscopy time (min)	0	22 ± 10	*P* < .01 ^b^
PVC suppression during ablation	80% (8/10)	55% ( 6/11)	*P* = .36^c^
Procedure complications	10% (1/10)	0% (0/11)	*P* = .48^c^
PVC recurrences follow‐up	30% (3/10)	27.3% (3/11)	*P* = 1^c^

Abbreviations: BMI, Body Mass index; LVEF, Left ventricular ejection fraction; PVC, Premature ventricular complex; RF, Radiofrequency.

^a^ Student's t test.

^b^ Mann‐Whitney U test.

^c^ Fisher's exact test.

## 4 DISCUSSION

The main finding of this study was that ZF catheter ablation of ASC‐PVC guided by ICE 3D‐EAM is a safe procedure, is no less effective than the conventional approach, and offers a significant reduction in radiation time.

This approach was found to provide various advantages, including: (a) direct visualization and integration of each aortic cusp and CAO in the EAM system; (b) real‐time measurement of the distance between ablation catheter tip and CAO in the navigation system; (c) real‐time ultrasound monitoring of ablation catheter tip contact during RF application; and (d) early detection of ablation complications such as steam pops^9^ or catheter dislocations.

ASC‐PVC ablations have been reported to represent 16% of ablations in outflow tracts.^10^ As noted above, CC has conventionally been used to ensure a safe distance (>5 mm) between ablation catheter tip and CAO.^1^ However, the catheterization must be repeated if the ablation catheter is repositioned during CC, and the integration of catheterization in mapping systems has been proposed to overcome this drawback.^10^ Nevertheless, lesser precision is obtained with closer proximity to the CAO, and another CC with new mapping system integration is required when the ablation catheter is repositioned, as reported in 25% of the patients studied by Jularic et al.^11^

Transesophageal echocardiography has been proposed as an alternative approach but has only been described in a few isolated cases.^12^ In addition, contrast injection through the ablation catheter has been proposed as a tool to visualize CAO, but the low flow of injected contrast limits the accuracy of CAO distance estimations.^13^ The use of ICE to guide ASC‐PVC ablation, as in the present study, resolves the problem of evaluating the distance from the catheter tip to ostia. 3D integration of the ICE image in the EAM system and real‐time monitoring of the relationship of catheter tip to CA ostia offers a comprehensive approach that does not require CC. A search of the literature revealed just one case series^14^ in which ICE was used for this purpose, although 9% of cases required CC to confirm the distance to CAO due to doubts about the position of the ablation catheter. In the present study, CC was not required in any patient treated with ICE 3D‐EAM‐guided ZF ablation, given that a clear safety distance could be established in all of these patients. Regarding acute success rate in the study group (80%), our results are similar than those published for Hoffmayer et al.^14^ (83%) and inferior than the Yamada and Jularic studies^1^, ^11^ (100% in both reports).

The feasibility of ablation procedures with ZF has been demonstrated in simple substrates such as supraventricular tachycardia or common atrial flutter.^6^, ^7^ They have reduced the exposure to radiation, which has been associated with acute and subacute skin lesions, oncological processes, cataracts, thyroid involvement, and other organ and system disorders.^15^, ^16^, ^17^ Interventions in cardiac electrophysiology should follow the principle “as low as reasonably achievable” (ALARA), and the ZF approach has been reported to reduce the risk of cancer and mortality by 96% in comparison to conventional fluoroscopy.^18^, ^19^ The emergence of ICE and its 3D integration has enhanced the visualization of adjacent anatomical structures and improved the efficacy and safety of complex procedures.^20^ To our best knowledge, no study has previously evaluated a completely ZF approach to ASC‐PVC ablation guided by ICE 3D‐EAM. The AVATAR Registry^21^ was a nonrandomized study that described ASC‐PVC ablation with ZF or near ZF approach and not guided by ICE 3D‐EAM. In this study CAO localization was performed with the ablation catheter without any guiding imaging technique. In our opinion, this method is not recommended due to the possibility of CA injury with fatal consequences. Furthermore, appropriate CAO localization with this technique has not been validated. In the present series, ASC‐PVC ablation with ZF approach was possible in all patients, and the ASC and both CAO were visualized by ICE in all cases. Furthermore, the application of ICE 3D‐EAM did not significantly increase the complication rate in comparison to conventional approach and we did not observe significant differences in the number of RF applications, acute success rate, or recurrence rate at 3 months follow‐up. Nevertheless, although not statistically significant, the duration of the procedure was increased in the ICE 3D‐EAM group. For this point, we believe that the main reason why we have a long procedure time is because the searching and delineation of the different anatomical structures that we must monitor during ablation, is sometimes difficult and time consuming.

### 4.1 Study limitations

First of all, this study is limited by its retrospective and nonrandomized design and small sample size. On the one hand, the ZF ICE 3D‐EAM procedures were done by experienced operators in this kind of interventions. These results may therefore not be directly replicable in other centers without enough ICE experience and further assessments are required for expanding these results. On the other hand, regarding origin classification of the ASC‐PVC in the conventional group, some PVC were classified as LCC origin; however, there can be a mismatch between the real localization of the PVC in the exact site of the cusp using angiography. Therefore, PVC origin may have been cataloged as LCC when in reality its origin is closer to LCC‐RCC. In addition, the ZF group did not include any patients with ASC‐PVC exclusively originating from the LCC, who might possibly have a greater need for CC due to the closer proximity to CAO. However, ICE 3D‐EAM offered a reliable and precise visualization of the LMCA ostium for evaluation of the safety distance. It should also be taken into account that 3 patients in the control group did not undergo ablation guided by contact force. Finally, a more prolonged follow‐up period is needed to evaluate long‐term success rates.

## 5 CONCLUSIONS

ZF catheter ablation of ASC‐PVC guided by 3D ICE‐EAM is an effective and safe procedure that reduces the radiation exposure of health care professionals and patients.

## CONFLICT OF INTEREST

No author has conflict of interest.

## AUTHOR CONTRIBUTIONS

Dr Sánchez‐Millán and Dr Gutiérrez‐Ballesteros: concept/design, data analysis/interpretation, drafting article, statistics. Rest of authors: critical revision of article and approval of article.

## Supporting information

Supplementary Material

Video S1

Video S2

Video S3

Video S4

## APPENDIX 1. Example of advancing the ICE catheter without fluoroscopy from femoral vein to right atrium.

After introducing the sheath in the common femoral vein, the ICE catheter is carefully advanced, always maintaining the view of the venous endovascular lumen in the ICE screen. If during advancing the probe any resistance is felt or the endovascular lumen view is lost, the probe is withdrawn a few centimeters and rotated in order to look for the endovascular lumen. After the venous lumen is seen, a slight anterior tilt is performed with the probe toward that area and the catheter is moved forward in order to advance the probe without any stop or resistance in the wall vessel and avoiding the entrance in a venous branch. Catheter withdrawal and redirection were typically required and this sequence of movements was repeated until reaching the heart.

## APPENDIX 2. Example of zero‐fluoroscopy catheter advancement retrogradely through the aorta via femoral arterial access to approach ASC.

In the arterial access, once the catheter has been introduced through the sheath, the catheter is advanced with gentle twisting movements. If any resistance is felt, the catheter is withdrawn and slight deflections and twists are made trying again to advance the catheter until it does so without resistance, reaching the descending aorta. At this point, the tip of the catheter is visible in the Carto System. Then, the catheter is advanced until reaching the anatomical area corresponding to the aortic arch. In a left anterior oblique view in the Carto System, a slight deflection of the catheter is made with the tip directed toward the ascending aorta (previously reconstructed with the Carto‐Sound) and is advanced gently. If no resistance is felt, the catheter is advanced until reaching the plane of the aortic valve. If any resistance is felt during the advancement of the catheter in the aortic arch, the catheter is usually pulled back and then, the anteflexion is increased and the catheter is progressed without resistance to the aortic valve plane.
