# Supplementary material for: Ablation with zero‐fluoroscopy of premature ventricular complexes from aortic sinus cusps: A single‐center experience
Source: J Arrhythm. 2021 Oct 3;37(6):1497–505. doi: 10.1002/joa3.12642 (PMC8637083; doi:10.1002/joa3.12642)
Supplement: Supplementary file 1 — Supplementary Material [file JOA3-37-1497-s004.docx]

**Supporting Document**

**Table S1.** Clinical characteristics of the zero-fluoroscopy group.

|  | **Age (y.o)** | **Sex** | **Ablation**  **Indication** | **Structural**  **heart disease** | **Ejection Fraction before ablation (%)** | **PVC density before ablation (% and absolute number)^*^** | **AAD**  **before**  **ablation** | **Previous ablation**  **Failure.** | **Follow-up recurrence.** | **PVC density after ablation (% and absolute number)^+^** | **AAD**  **after**  **ablation** |
| --- | --- | --- | --- | --- | --- | --- | --- | --- | --- | --- | --- |
| **Patient 1** | 47 | Male | Palpitations | HCM^A^ | 63 | 14 %  8329 PVC | No | Yes | Yes | 25 %  24810 PVC | No |
| **Patient 2** | 31 | Female | Palpitations | No | 55 | 34.50 %  31403 PVC | BB | Yes | Yes | 19 %  31403 PVC | Flecainide |
| **Patient 3** | 55 | Female | Low Ejection Fraction | TCM^B^ | 34 | 20.50 %  18123 PVC | BB | Yes | No | 0 %  0 PVC | No |
| **Patient 4** | 67 | Male | Low Ejection Fraction | TCM^B^ | 40 | 26 %  23516 PVC | BB | No | No | 2.9 %  2623 PVC | BB |
| **Patient 5** | 43 | Male | Palpitations | No | 63 | 21.20 %  40190 PVC | BB | Yes | No | 0.01 %  10 PVC | No |
| **Patient 6** | 14 | Male | Palpitations | No | 67 | 21 %  23848 PVC | No | No | No | 0.98 %  988 PVC | No |
| **Patient 7** | 67 | Female | Palpitations | TCM^B^ | 44 | 22.80 %  20719 PVC | BB | No | No | 0.01 %  36 PVC | No |
| **Patient 8** | 55 | Male | Palpitations | No | 66 | 21.30 %  17091 PVC | BB | No | No | 0 %  0 PVC | No |
| **Patient 9** | 60 | Male | Palpitations | No | 53 | 38.90 %  39377 PVC | BB | No | No | 0 %  0 PVC | No |
| **Patient 10** | 48 | Female | Low Ejection Fraction | AVC^C^ | 43 | 28.60 %  30406 PVC | BB | No | Yes | 26.4 %  28067 PVC | BB |

HCM: Hypertrophic cardiomyopathy. B. TCM: Tachycardia-induced cardiomyopathy. C: Arrhythmogenic cardiomyopathy. PVC: Premature ventricular complex. * 24-hour Holter ECG prior to ablation. ^+^ 24- hour Holter -ECG 3 months after ablation. BB: Beta-blocker.

**Table S2.** Procedure-related variables in control group

|  | **PVC ablation site** | **Mapping catheter/ Ablation Catheter** | **Ablation success** | **Procedure**  **Time (min)** | **RF ablation time (s)** | **Number**  **of RF Lesions** | **Fluoroscopy**  **Time (min)** | **Right CAO distance (mm)** | **Left Main CAO distance**  **(mm)** | **Complications** |
| --- | --- | --- | --- | --- | --- | --- | --- | --- | --- | --- |
| **Patient 1** | NCC | Thermocool/  Thermocool | Yes | 165 | 126 | 2 | 27 | 12.5 | — | No |
| **Patient 2** | LCC | Tacticath/  Tacticath | Yes | 220 | 67 | 1 | 23 | — | 8 | No |
| **Patient 3** | LCC | Thermocool/  Thermocool | Yes | 265 | 225 | 5 | 29 | — | 21 | No |
| **Patient 4** | LCC | Thermocool/  Thermocool | No | 325 | 557 | 25 | 43 | — | 7.5 | No |
| **Patient 5** | LCC | PentaRay/  Smart-Touch | No | 160 | 229 | 9 | 26 | — | 12.3 | No |
| **Patient 6** | RCC | Smart-Touch/  Smart-Touch | Yes | 217 | 629 | 13 | 18 | 11 | — | No |
| **Patient 7** | LCC-RCC | Smart-Touch/  Smart-Touch | No | 310 | 418 | 13 | 15 | 25,5 | 11.1 | No |
| **Patient 8** | NCC | PentaRay/  Smart-Touch | Yes | 154 | 119 | 2 | 9 | 20 | — | No |
| **Patient 9** | LCC | PentaRay/  Smart-Touch | Yes | 285 | 267 | 5 | 10 | — | 19.1 | No |
| **Patient 10** | RCC | Smart-Touch/  Smart-Touch | No | 210 | 264 | 4 | 20 | 16 | — | No |
| **Patient 11** | LCC | PentaRay/  Smart-Touch | No | 205 | 309 | 5 | 20 | — | 14.5 | No |

PVC: Premature ventricular complex. RF: Radio-frequency. LCC: Left coronary cusp. NCC: Non-coronary cusp. RCC: Right coronary cusp. LCC-RCC Between right and left coronary Cusp. CAO: Coronary artery ostium.

**Video S1**

First part of the video shows the delineation and 3D reconstruction of CAO using CARTOSOUND®. The second part of the video shows how to cross the ablation catheter through the aortic valve using the 3D reconstruction of ASC and CAO without fluoroscopy. CAO: coronary artery ostiums. ASC: aortic sinus cusps

**Video S2**

Example of ZF catheter ablation of ASC-PVC. The origin of the PVC in this case was in LCC-RCC junction and the ablation catheter in the successful spot is shown. There is a safe distance from the tip of the ablation catheter to the CAO clearly visualized in CARTOSOUND® reconstruction. ZF: zero-fluoroscopy. ASC-PVC: aortic sinus cusps premature ventricular complexes. LCC-RCC: left coronary cusp-right coronary cusp. CAO: coronary artery ostiums.

**Video S3.**

Example of the advance of the ICE catheter without fluoroscopy from femoral vein to right atrium. The ICE catheter may be freely advanced throughout the vessels and cardiac structures if the leading edge has an echo-free space. Catheter withdrawal and redirection are typically all that is required to enter the right atrium. ICE: intracardiac echocardiography.

**Video S4.**

Example of zero-fluoroscopy catheter advancement retrogradely through the aorta via femoral arterial access to approach ASC. The left anterior oblique in the navigation system is used to guide the tip of the catheter towards the aortic arch and ascending aorta and reach the aortic valve plane. ASC: aortic sinus cusps
